# Physiological and behavioral parameters of pain and stress in mares during and after transvaginal ultrasound-guided follicular aspiration

**DOI:** 10.3389/fvets.2025.1574351

**Published:** 2025-04-09

**Authors:** Emma Van den Branden, Matthieu Salamone, Klaartje Broothaers, Sofie Peere, Ellen Polfliet, Manon Dewulf, Glenn Van Steenkiste, Gunther van Loon, Katrien Smits, Jan Govaere

**Affiliations:** ^1^Department of Internal Medicine, Reproduction, and Population Medicine, Faculty of Veterinary Medicine, Ghent University, Merelbeke, Belgium; ^2^Division of Animal and Human Health Engineering, Department of Biosystems, KU Leuven, Leuven, Belgium; ^3^Equine Cardioteam Ghent, Department of Internal Medicine, Reproduction, and Population Medicine, Faculty of Veterinary Medicine, Ghent University, Merelbeke, Belgium

**Keywords:** equine, assisted reproduction, ovum pick-up, animal welfare, stress

## Abstract

**Introduction:**

The use of transvaginal ultrasound-guided follicle aspiration (TVA) for oocyte collection has become a widely used procedure in horses for *in vitro* embryo production (IVEP). The TVA procedure is characterized by various manipulations, which are physical restraint, perineal preparation, transrectal palpation, insertion of an intravaginal device, and needle punctures. These repeated transvaginal and transovarian punctures have raised concerns about their potential effects on mare welfare. Our study aimed to investigate the effects of TVA manipulations, and especially puncturing, on pain and stress in mares in a commercial set-up.

**Methods:**

Therefore, eight mares were subjected to three TVA procedures: two with puncturing (P^+^) and one without (P^−^, control). Before, during and after all TVA procedures, blood was collected for serum glucose, lactate and cortisol levels, and facial pain scores were assessed. An electrocardiogram (ECG) was recorded for each mare before and during the procedure to measure heart rate (HR) and heart rate variability (HRV). Parameters in response to manipulations and puncturing were analyzed using linear mixed effect models for each outcome variable at different timepoints during the procedure.

**Results:**

Results revealed that puncturing during TVA did not significantly influence serum metabolite levels, facial pain scores, HR, or HRV over the complete procedure (*p* > 0.05). Notably, HR did not increase at the moment of puncturing (*p* > 0.05), and no significant changes in HRV parameters between P^+^ and P^−^ were detected (*p* > 0.05). Both P^+^ and P^−^ procedures triggered significant increases in cortisol and lactate levels, facial pain scores, and HR during restraint in stocks and perineal preparation compared to the day before and the day after TVA (*p* < 0.05). Interestingly, even without puncturing, manipulations in the P^−^ procedure were sufficient to induce significant elevations in metabolite levels and facial pain scores compared to the day before and after (*p* < 0.05).

**Discussion:**

These results indicate that the TVA procedure induces stress responses in mares, predominantly associated with all manipulations specific to TVA, while the effect of puncturing itself was minimal. All effects were acute, with parameters returning to baseline when measured 24 h later.

## 1 Introduction

In horses, *in vitro* embryo production (IVEP) through transvaginal ultrasound-guided follicle aspiration (TVA) followed by intracytoplasmic sperm injection has emerged in the early 1990s as an innovative breeding technique, offering a more advanced alternative to traditional embryo flushing (1–4). TVA involves aspirating follicles directly from the ovaries using an ultrasound-guided needle passed through the vaginal wall, allowing for oocyte retrieval from mares. The commercial demand for these *in vitro* produced embryos among breeders and owners has grown over the past decade, driven by the improvement in the production of transferable blastocysts and pregnancy success rates (1, 5, 6). According to the International Embryo Transfer Society, *in vitro* produced embryos have outnumbered those produced by embryo flushing in Europe (1, 7). The efficiency, versatility, and high pregnancy rates achieved with *in vitro* produced embryos, along with easier embryo cryopreservation and facilitation of international trade, could explain why it now outperforms traditional breeding methods (1, 2, 6, 8, 9).

Despite its benefits, it is crucial to highlight that TVA involves various manipulations, including transrectal palpation, insertion of an intravaginal device, and multiple needle punctures into the vagina wall, peritoneum, and ovaries. Additionally, the same mare often undergoes multiple TVA sessions, sometimes as frequently as every 2 weeks. In women, the TVA procedure is considered painful (10, 11), with the level of pain associated with the diameter of the TVA needle (12, 13), suggesting the need to recognize and minimize discomfort in animals. Limited data exist on the well-being of mares during and after TVA, as most studies focus on health and fertility (8, 14–16). Currently, only a few studies, mainly limited to livestock, have investigated the welfare of animals undergoing reproductive interventions, and specifically TVA. In cattle, TVA induces moderate stress, but repeated procedures do not harm well-being (17–20). In sows, stress is temporary, with feeding drive outweighing discomfort (21). In mares, transrectal ultrasound causes stress (22) and needle puncturing during TVA increases heart rate (23).

Assessing pain and stress in horses is challenging due to the involvement of different physiological systems (e.g., neuroendocrine and cardiovascular) and the stoic nature of prey animals (24, 25). Stress is broadly defined as a disruption of homeostasis or an animal’s inability to cope with its environment (19, 26). As no single parameter is highly specific to pain and stress, an integrated approach that monitors a variety of indicators, including physiological and behavioral parameters, has traditionally been used (24, 25). The behavioral and physiological parameters used in this study were motivated by the two main pathways, the hypothalamic–pituitary–adrenal cortex (HPA) axis and the sympathetic-adrenal medulla (SAM) axis, activated by the equine stress response (27–29). Previous studies in livestock typically evaluated stress during TVA by monitoring behavioral responses, measuring cortisol and ACTH levels, assessing milk production, and tracking HR (18, 19, 21, 23). Other valuable markers to monitor stress and pain in animals are validated pain scales, lactate and glucose levels, and heart rate variability (HRV). Validated pain scales offer a more objective method to monitor pain over time (30), serum lactate and glucose levels are well-established indicators of acute stress (30–33), and HRV reflects the balance between sympathetic and parasympathetic tone and is thus commonly used to assess the autonomic nervous system’s stress response (34). To our knowledge, no previous study in horses investigated the effect of TVA manipulations and puncturing on serum lactate and glucose levels, and HRV.

Since animal welfare is becoming a priority in breeding programs, it is crucial to assess whether the TVA procedure affects the well-being of mares. The main concern with TVA is the repeated transvaginal and transovarian punctures, which are both invasive and specific to this procedure. We hypothesized that all manipulations inherent to the TVA procedure may cause discomfort and stress, with puncturing during TVA being the most sensitive step. Therefore, the aim of the present study was to evaluate the effects of all TVA-related manipulations, and puncturing in particular, on pain and stress in mares in a commercial program, including the standard medication protocol. This was achieved by monitoring HR, HRV, facial expressions, glucose, lactate and cortisol levels during and between procedures.

## 2 Materials and methods

### 2.1 Study design

Eight clinically healthy mares of mixed breeds, aged between 5 and 18 years old, were included in this study. All mares were housed in individual boxes with *ad libitum* access to water and hay. The animals were familiar with the facility, its staff, and the protocols used in the procedure. All experimental protocols were approved by the Ethical Committee of Ghent University (reference number 2023–075).

One month prior to the study, a 24-h electrocardiogram (ECG) was recorded using a Televet100 recording system (Engel Engineering Services GmbH, Germany) to establish the normal cardiovascular parameters, including HR and HRV, for each mare in the stable.

**Figure 1:**
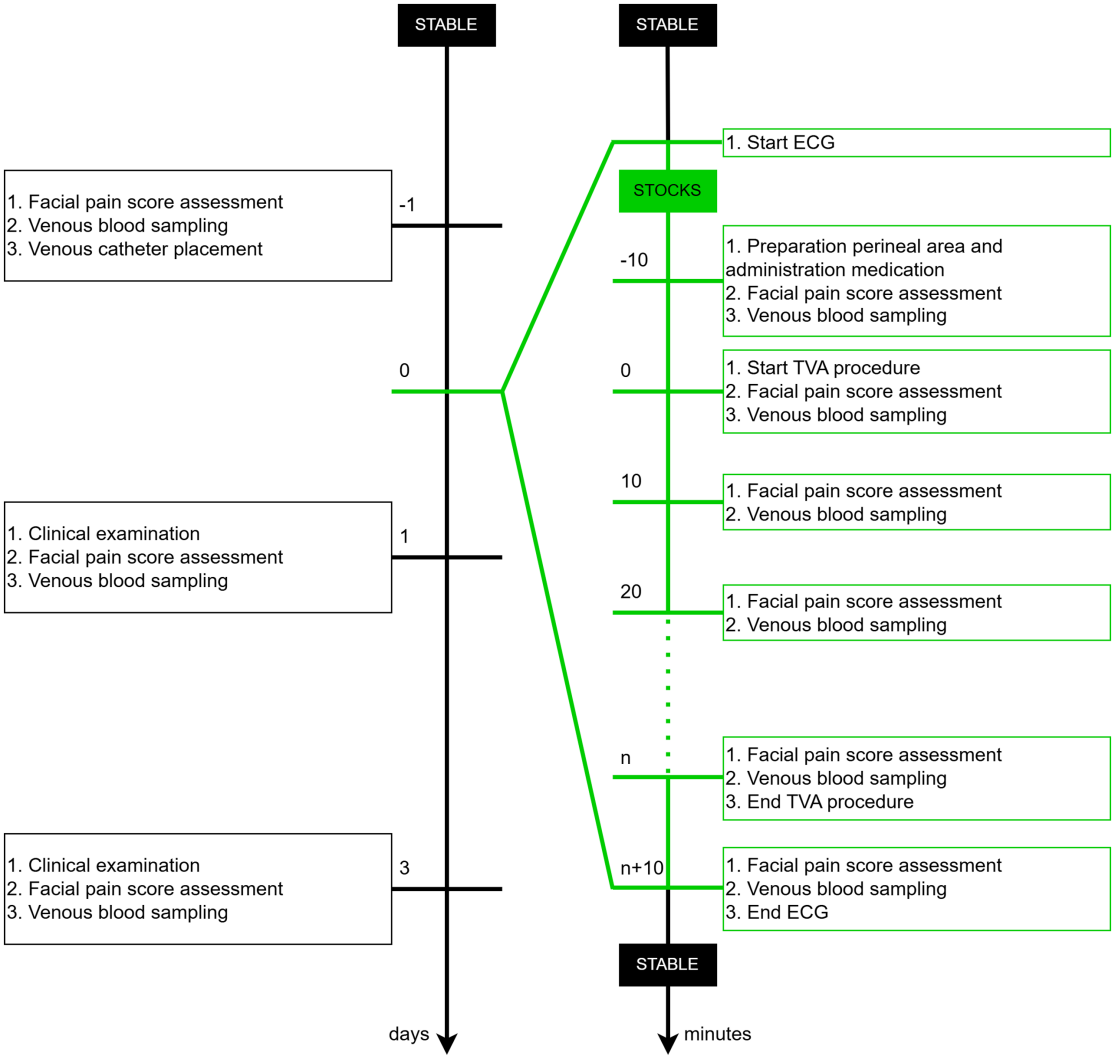
Timeline of the experimental protocol depicting key procedures in chronological order across days and minutes.

Each mare (Horse A-H) underwent three sessions (Replicate 1–3), with a 14-day interval between each session. The first two TVA sessions involved puncturing (P^+^), whereas the third session, without puncturing, served as a control (P^−^). The sessions were conducted between January and March 2024. All experiments were performed between 08 h00 and 12 h00 to account for the circadian rhythm in cortisol release (35). The protocol for each session is outlined below (Figure 1), with detailed methods described further:

- Day before TVA: A facial pain score assessment and blood tests were performed. An intravenous catheter (Mila IV catheter 14G 2.00 × 130 mm; Mila International, Kentucky, United States) was placed in the jugular vein.
- Day of TVA: The ECG recording system was fitted to each mare in the cardiology examination room to continuously monitor HR and HRV. Once the recording system was in place, the mares were moved to the stocks for the TVA procedure. Ten minutes before the start of the TVA procedure, while the mare was in the stocks and after the medication was administered, the first blood sample was collected and the facial pain score was assessed (T_−10_). The start of the procedure was defined as the moment the intravaginal probe was inserted and the ovary was manipulated (T_0_). Subsequent blood samplings and facial pain score evaluations were conducted at 10-min intervals throughout the procedure, continuing until 10 min after its completion, while the mare remained in the stocks (T_10_, T_20_, T_30_, T_40_).
- Post-TVA follow-up: On Day 1 and 3 post-TVA, a comprehensive clinical examination (respiratory rate, HR, and body temperature), facial pain score assessment, and blood sampling were repeated for each mare.

### 2.2 Description of critical methods

#### 2.2.1 Transvaginal ultrasound-guided follicular aspiration

The mares were restrained in the stocks within the examination room for the TVA procedure. The tail was wrapped and secured, the rectum was emptied, and the perineal region was cleaned thoroughly at least three times using 7.5% polyvidone iodine antiseptic soap (Iso-Betadine Germicidal Soap 7.5%; Viatris Healthcare, Pennsylvania, United States). Each mare was administered following medication prior to the TVA procedures:

- 15 μg/kg detomidine IV (Domidine, 10 mg/mL detomidinehydrochloride; Dechra, The Netherlands),
- 10 mg butorphanol IV (Dolorex, 10 mg/mL butorfanol (tartraat); MSD Animal Health, Belgium),
- 1 mg/kg flunixine meglumine IV (Emdofluxin, 50 mg/mL flunixine meglumine; Emdoka, Belgium),
- 21,000 IE/kg procaïne benzylpenicilline IM (Peni-Kel, 300,000 IE/mL procaïne benzylpenicilline; Kela, Belgium),
- 0.2 mg/kg butylhyoscine bromide IV (Buscopan Compositum ad us. Vet., 500 mg/mL natriummetamizol +4 mg/mL butylhyoscine bromide; Boehringer Ingelheim, Germany).

A constant rate infusion of 2 mL/100 kg was administered, consisting of 19 mL isotonic sodium chloride solution and 1 mL Domidine (10 mg/mL detomidinehydrochloride; Dechra, The Netherlands). Additional doses of detomidine [5 μg/kg detomidine IV (Domidine, 10 mg/mL detomidinehydrochloride; Dechra, The Netherlands)] were administered if excessive movement interfered with the operability of the manipulator during the procedure, ensuring the safety of the animal and personnel, and facilitating the completion of the TVA.

After aseptic preparation of the perineal area, a transvaginal ultrasound probe (Esaote EC123 Intra CavityA; Esaote, Italy), with a frequency range of 5.0–7.5 MHz, was fitted with a custom needle guide and inserted into the vagina. The ovary was then manipulated and fixed by rectal palpation against the vaginal wall by an experienced veterinarian. During the P^+^ procedure, a 12 G x 25 inch double-lumen oocyte aspiration needle (Minitube, Germany) was inserted through the transducer needle guide and once a follicle was visualized, the needle was introduced into the ovary through the vaginal wall, and the follicle was aspirated. All visible follicles were punctured, aspirated, and flushed 10 times with 1–3 mL of prewarmed flushing medium (Equiplus; Minitube, Germany) using a vacuum pump (Minitube, Germany). During aspiration, the follicle walls were scraped by rotating the needle. The P^+^ procedure ended when all follicles were aspirated.

During the P^−^ procedure, the mares underwent the same procedure with identical manipulations (including preparation of the perineum, insertion of the intravaginal ultrasound probe, and rectal manipulation in search of follicles and thus with traction on the broad ligament), but no needle was introduced. The mares received the same medications but no antibiotics; instead 7 mL/100 kg physiological saline IM was administered as a placebo (NaCl 0.9% Ecoflac Plus; B. Braun, Germany). The P^−^ procedure ended after 17 min of rectal manipulation on each ovary, corresponding to the expected duration of a TVA.

#### 2.2.2 Catheter placement

Handling and restraining animals during blood collection can be stressful and can lead to increased cortisol levels; therefore, an intravenous catheter (Mila IV catheter 14G 2.00 × 130 mm; Mila International, Kentucky, United States) was placed the day before to avoid this potential confounding factor. To place the intravenous catheter into the jugular vein, the jugular groove was located and clipped at the midpoint of the middle third of the jugular vein. The clipped area was aseptically prepared using alternating scrubs of chlorhexidine and alcohol. A volume of 1–2 mL of 2% lidocaine was administered subcutaneously at the planned insertion site to provide local anesthesia and desensitize the area. The jugular vein was occluded manually at the thoracic inlet and the catheter was introduced at a 30–45° angle, oriented caudally in alignment with the direction of venous blood flow. Upon observing a flash of blood in the catheter hub, the stylet was removed, and the catheter was advanced fully into the vein. A sterile perfusion line with a three-way stopcock was attached to the catheter to allow for fluid administration and secure port access. The catheter was then sutured to the skin to ensure stability and prevent dislodgement.

#### 2.2.3 Blood sampling and serum metabolite measurements

Blood was collected aseptically into serum and fluoride/oxalate tubes from the jugular vein before and after the procedure, and from the catheter during the procedure. Samples were stored at 4°C until analysis. Lactate and glucose levels were measured in-house using a RAPIDPoint 405 Blood Gas Analyzer (Siemens Healthcare Diagnostics, Germany). Cortisol levels were sent the same day to an external laboratory for analysis using electrochemiluminescence immunoassays with the Elecsys Cortisol Assay Kit (Roche Diagnostics, Germany; catalog number 11875116). The assay has a detection range of 0.5–1750 nmol/L, with intra-assay and inter-assay coefficients of variation below 5%.

#### 2.2.4 Facial pain score

The Equine Utrecht University Scale for Facial Assessment of Pain (EQUUS-FAP), as described by van Loon and Van Dierendonck (30), was used to assess facial pain scores. This was consistently performed by the same veterinarian. This multifactorial descriptive scale is specifically designed to monitor acute visceral pain in horses and is based on 9 parameters, describing different elements of facial expression (head, eyelids, focus, nostrils, corners mouth/lips, muscle tone head, flehming and/or yawning, teeth grinding and/or moaning, and ears). Each of the nine parameters can be scored from 0 to 2, leading to a total pain score ranging from 0 (no signs of pain) to 18 (maximal pain score) (Table 1).

**Table 1:** Score sheet of the Equine Utrecht University Scale for Facial Assessment of Pain (EQUUS-FAP) (29).

Data	Categories	Score
Head	Normal head movement/interested in environment	0
	Less movement	1
	No movement	2
Eyelids	Opened, sclera can be seen in case of eye/head movement	0
	More opened eyes or tightening of eyelids. And edge of the sclera can be seen 50% of the time	1
	Obviously more opened eyes or obvious tightening of the eyelids. Sclera can be seen >50% of the time	2
Focus	Focused on environment	0
	Less focused on environment	1
	Not focused on environment	2
Nostrils	Relaxed	0
	A bit more opened	1
	Obviously more opened, nostrils flaring and possibly audible breathing	2
Corners mouth/lips	Relaxed	0
	Lifted slightly	1
	Obviously lifted	2
Muscle tone head	No fasciculations	0
	Mild fasciculations	1
	Obvious fasciculations	2
Flehming and/or yawning	Not seen	0
	Seen	2
Teeth grinding and/or moaning	Not heard	0
	Heard	2
Ears	Position: orientation toward sound/clear response with both ears or ear closest to source	0
	Delayed/reduced response to sounds	1
	Position: backwards/no response to sounds	2
Total		…/18

#### 2.2.5 Heart rate and heart rate variability

In all mares, ECG recordings were obtained using a Televet100 recording system as described by Broux et al. (36). Four self-adhesive electrodes were placed underneath a girth (Mainat Vet, Spain). The right arm electrode was positioned 15 cm right of the withers, the left leg electrode caudal to the left elbow on the thorax, and the left arm electrode 10 cm above the left leg electrode resulting in a modified base-apex configuration. A reference electrode was placed on the left side of the withers. All electrodes were connected to the recording device, placed in the girth.

ECG data were processed with custom Matlab (Mathworks, MA, United States) scripts for filtering, normalization and QRS complex detection (37). Automatic QRS markers were manually corrected. Bifid P waves were identified within specific windows set by Q onset and T wave offset, considering amplitude, slope, morphology, and prominence to detect P onset (Pon), P1 peak and P2 peak. Ten HRV parameters were calculated from the different P wave indices and RR intervals over 2 min time windows. Considering the acceptable standards for ECG recordings, the usage of 2-min intervals for determining the 10 HRV parameters, specifically the SDNN parameter, is shorter than the 5-min standard. However, the choice of shorter segments was made in the context of our brief intervention.

Four time domain parameters were determined: SDNN (standard deviation of the RR intervals), RMSSD (root mean squared successive differences in RR intervals), pNN50 (proportion of intervals above 50 ms) and TRI (triangular index; integral of RR interval histogram divided by height of the histogram); four frequency domain parameters were calculated: very low- (VLF, 0.0033–0.04 Hz), low- (LF, from 0.04–0.15 Hz), and high-frequency (HF, 0.15–0.4 Hz) components, and LF/HF ratio (the ratio of the low frequency band over the high frequency band); two nonlinear parameters were determined: SD1 and SD2 (standard deviation of the Poincaré plot perpendicular to and along the line of identity, respectively). For a comprehensive overview of HRV variables, including their baseline values and variation ranges, please refer to Supplementary Table 1.

### 2.3 Statistical analysis of data

All statistical analyses were performed using R (38), version 4.4.0 including packages “tidyverse” (39), “skimr” (40), “lme4” (41), “lmerTest” (42), “performance” (43), “sjPlot” (44), “ggforce” (45), “emmeans” (46), “ggpubr” (47), “simr” (48). All statistical analyses including code scripts can be consulted at https://doi.org/10.5281/zenodo.14245595.

Serum metabolite levels, facial pain scores, HR, HRV and sedation levels in response to puncturing (P^+^/P^−^) were analyzed using linear mixed effect models for each outcome variable. For serum metabolite levels and facial pain scores, puncturing and time were included as fixed effects, horse as a random intercept, and replicate as a random slope. For HR, two models were made. In the first HR model, housing environment (stable or stocks) and sedation were included as fixed effects, and horse as a random intercept. In the second HR model, sedation, intravaginal device insertion, rectal manipulation, and puncturing were included as fixed effects, horse as a random effect, and replicate as a random slope. HRV parameters were analyzed in a specific time window between the insertion and removal of the intravaginal device. To normalize these parameters, we applied a natural logarithm transformation to SDNN, RMSSD, TRI, SD1, and SD2, and a square root transformation to pNN50, VLF, LF, HF, and the LF/HF ratio. For HRV analysis, puncturing was included as a fixed effect, horse as a random intercept, and replicate as a random slope. For sedation levels, the total dosage of detomidine administered during the procedure was calculated per kilogram body weight per minute. Next, a natural logarithm transformation was applied. Puncturing was included as a fixed effect and Horse as a random effect. Statistical significance was defined at *p*-value ≤0.05.

## 3 Results

### 3.1 Descriptive analysis

Baseline measurements taken 24 h before all TVA procedures showed average cortisol levels of 5.3 μg/dL [normal range: 1.5–7.0 μg/dL (49–51)], lactate levels of 0.7 mmol/L [normal range: 0.0–1.5 mmol/L (52)], glucose levels of 90 mg/dL (normal range: 75–115 mg/dL (53)), and facial pain scores averaging 1.25 [normal range: 0–3 (30)]. Each mare underwent a median of six transovarian punctures (range: 5–8) per P^+^ procedure, with a median of 11 follicles aspirated (range: 7–32), and an oocyte recovery rate of 56% ± 18%. The average time per TVA procedure, counted from the insertion to the removal of the intravaginal device, was 35 min. The longest TVA procedure lasted 56 min; however, since it involved only one procedure and given the study’s model design, times over 50 min were excluded from statistical analysis. All clinical examinations performed on Day 1 and 3 after TVA procedure were within normal physiological levels.

### 3.2 Model performances

#### 3.2.1 Metabolites

**Figure 2:**
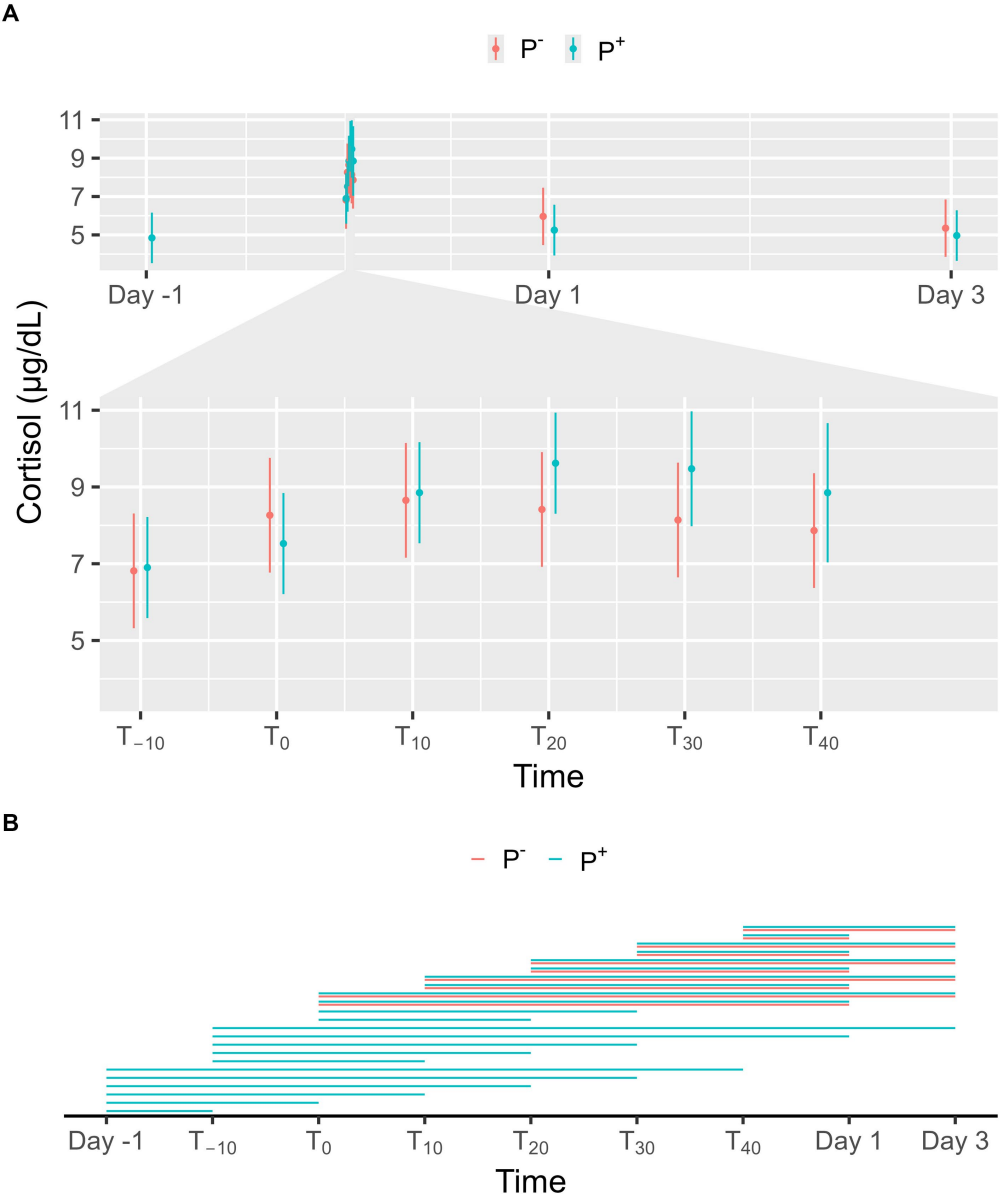
**(A)** The interaction between time and puncturing in the linear regression model with cortisol (μg/dL) as the dependent variable is shown. The least-squares means (LSM) of puncturing are plotted in 2 categories: no puncturing (P^−^) during transvaginal ultrasound-guided follicle aspiration (TVA) (red) and puncturing (P^+^) during TVA (blue). The bars represent the 95% CI of these effects. Significant differences (*p* < 0.05) found in a pairwise comparison between the LSM within each timepoint are indicated with “*.” **(B)** Pairwise comparison within the P^+^ (blue) and P^−^ (red) procedure over the different timepoints are plotted in this panel. A line between two timepoints indicates a significant difference (*p* < 0.05).

No significant effect on cortisol levels was found for P^+^ when compared to P^−^ in the main effect, but a significant interaction effect was found between puncturing and time. At individual timepoints, no differences were observed between P^+^ and P^−^, as shown in Figure 2A. For both P^+^ and P^−^, cortisol levels on Day 1 and 3 were significantly lower than during the procedure (T_−10_, T_0_, T_10_, T_20_, T_30_, and T_40_). Similarly, P^+^ showed significantly lower cortisol levels on Day-1 compared to procedural levels. Within the P^+^ procedure, cortisol levels at T_20_ and T_30_ were higher than at other timepoints. Additional significant interaction effects were found at T_10_, T_20_, and T_30,_ and are shown in Figure 2B.

**Figure 3:**
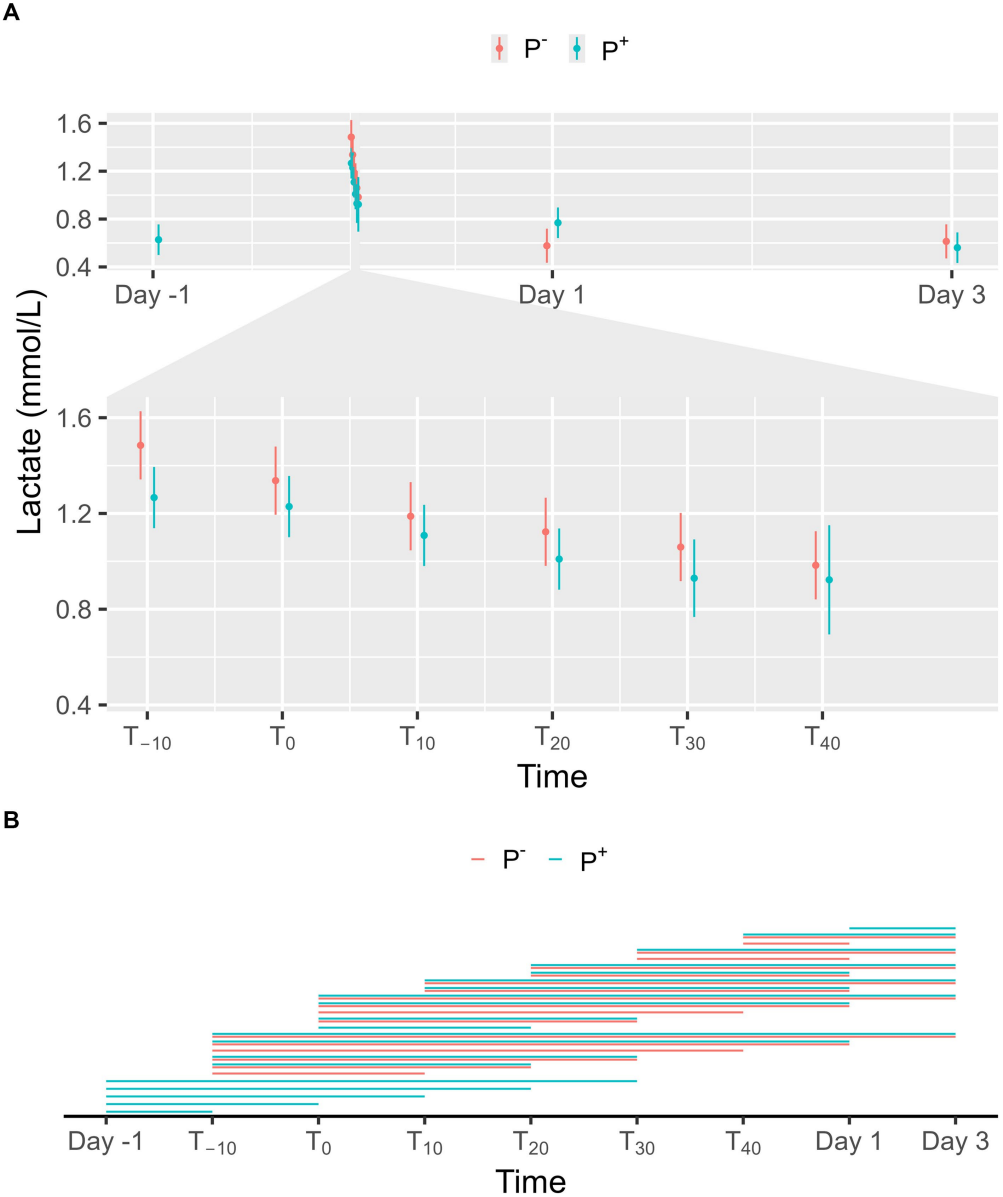
**(A)** The interaction between time and puncturing in the linear regression model with lactate (mmol/L) as the dependent variable is shown. The least-squares means (LSM) of puncturing are plotted in 2 categories: no puncturing (P^−^) during transvaginal ultrasound-guided follicle aspiration (TVA) (red) and puncturing (P^+^) during TVA (blue). The bars represent the 95% CI of these effects. Significant differences (*p* < 0.05) found in a pairwise comparison between the LSM within each timepoint are indicated with “*.” **(B)** Pairwise comparison within the P^+^ (blue) and P^−^ (red) procedure over the different timepoints are plotted in this panel. A line between two timepoints indicates a significant difference (*p* < 0.05).

No significant effect on lactate levels was found for P^+^ when compared to P^−^ in the main effect, but a significant interaction effect was found between puncturing and time. No difference between P^+^ and P^−^ was found at any specific timepoint, as shown in Figure 3A. For P^−^, lactate levels on Day 1 and 3 were significantly lower than during the procedure (T_−10_, T_0_, T_10_, T_20_, T_30_, and T_40_). For P^+^, lactate levels were significantly lower on Day-1 (except at T_40_), on Day 1 (except at T_30_ and T_40_), and on Day 3 compared to procedural levels. Additionally, lactate levels for P^+^ were significantly higher on Day 1 compared to Day 3. During the procedure, lactate levels at T_−10_ and T_0_ were significantly higher than at other timepoints for both P^+^ and P^−^. Other significant interaction effects were found at T_−10_, T_0_, T_30_, and T_40_ and are shown in Figure 3B.

**Figure 4:**
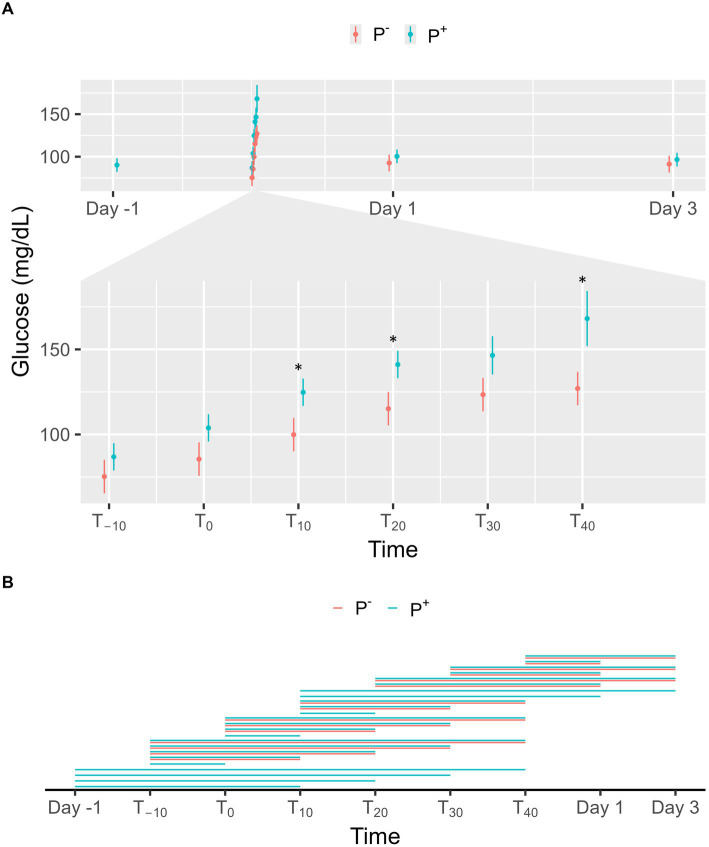
**(A)** The interaction between time and puncturing in the linear regression model with glucose (mg/dL) as the dependent variable is shown. The least-squares means (LSM) of puncturing are plotted in 2 categories: no puncturing (P^−^) during transvaginal ultrasound-guided follicle aspiration (TVA) (red) and puncturing (P^+^) during TVA (blue). The bars represent the 95% CI of these effects. Significant differences (*p* < 0.05) found in a pairwise comparison between the LSM within each timepoint are indicated with “*.” **(B)** Pairwise comparison within the P^+^ (blue) and P^−^ (red) procedure over the different timepoints are plotted in this panel. A line between two timepoints indicates a significant difference (*p* < 0.05).

No significant effect on glucose levels was found for P^+^ when compared to P^−^ in the main effect, but a significant interaction effect was found between puncturing and time. At T_10_, T_20_ and T_40_, glucose levels were significantly higher in P^+^ compared to P^−^, as shown in Figure 4A. For P^−^, glucose levels on Day 1 and 3 were significantly lower than at T_20_, T_30_, and T_40_. For P^+^, glucose levels on Day-1, Day 1, and Day 3 were significantly lower than at T10, T20, T30, and T40. During the procedure, glucose levels at T_−10_, T_0_, and T_10_ were significantly lower than at later timepoints for both P^+^ and P^−^. Additional significant interaction effects were found for T_−10_, T_0_, and T_10_ and are shown in Figure 4B.

#### 3.2.2 Facial pain score

**Figure 5:**
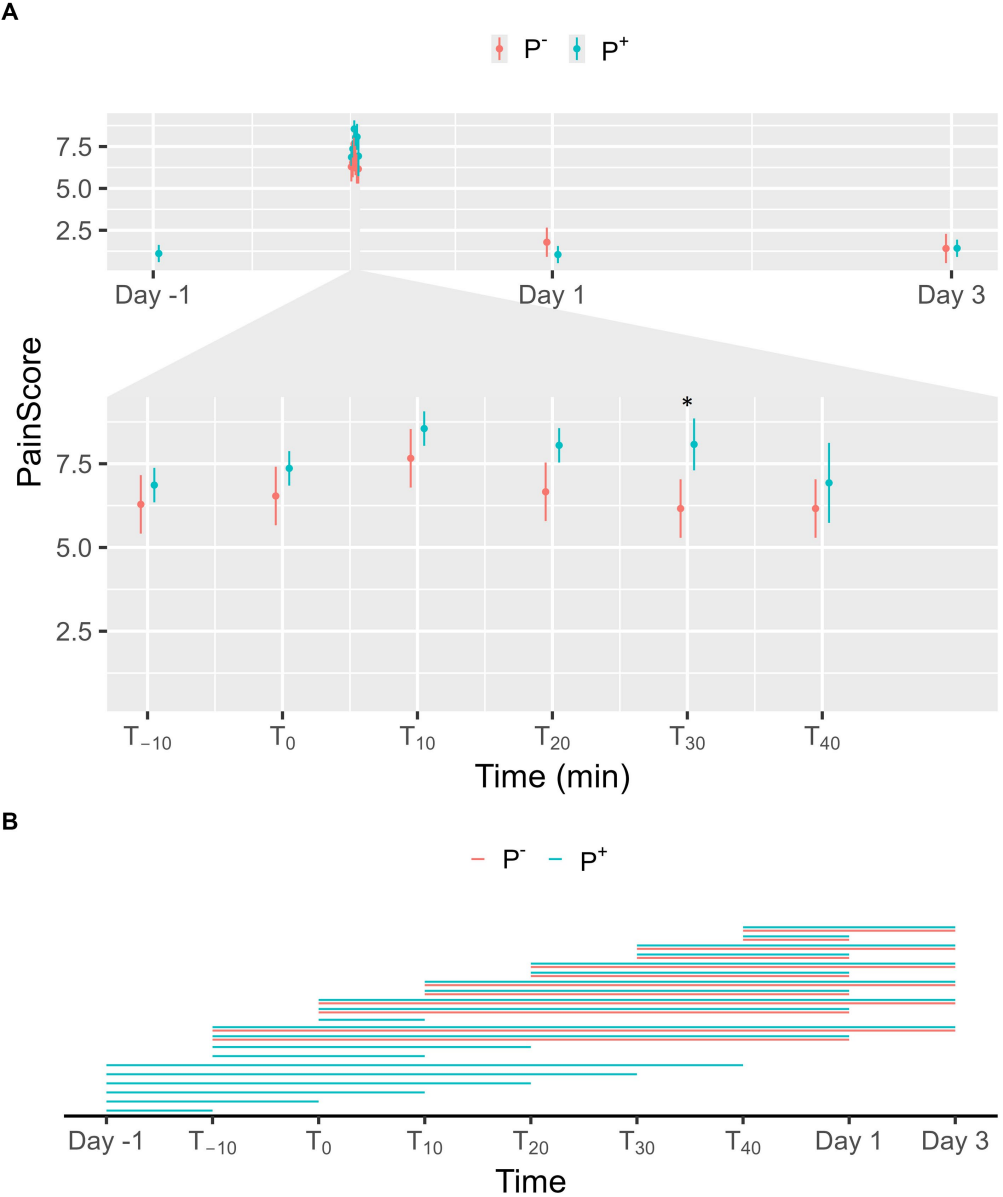
**(A)** The interaction between time and puncturing in the linear regression model with facial pain score [according to Equine Utrecht University Scale for Facial Assessment of Pain (EQUUS-FAP)] as the dependent variable is shown. The least-squares means (LSM) of puncturing are plotted in 2 categories: no puncturing (P^−^) during transvaginal ultrasound-guided follicle aspiration (TVA) (red) and puncturing (P^+^) during TVA (blue). The bars represent the 95% CI of these effects. Significant differences (*p* < 0.05) found in a pairwise comparison between the LSM within each timepoint are indicated with “*.” **(B)** Pairwise comparison within the P^+^ (blue) and P^−^ (red) procedure over the different timepoints are plotted in this panel. A line between two timepoints indicates a significant difference (*p* < 0.05).

No significant effect on facial pain scores was found for P^+^ when compared to P^−^ in the main effect, but a significant interaction effect was found between puncturing and time. As shown in Figures 5A, a significant difference was found at T_30_ with facial pain scores being higher in P^+^ than in P^−^. For P^−^, facial pain scores on Day 1 and 3 were significantly lower than during the procedure (T_−10_, T_0_, T_10_, T_20_, T_30_, and T_40_). For P^+^, facial pain scores were significantly lower on Day-1, on Day 1, and on Day 3 compared to procedural levels. During the procedure, facial pain scores at T_−10_ and T_0_ were significantly lower than at later timepoints for P^+^. Additional significant interaction effects were found for T_−10_ and T_0_ and are shown in Figure 5B.

#### 3.2.3 Heart rate and heart rate variability

An estimated increase of 22.6 bpm in HR was found when the mares were placed in the stocks. Sedation significantly decreased HR, with an estimated decrease of 8.5 bpm. No effect was observed on HR for intravaginal device insertion, rectal manipulation, and puncturing. No effect on SDNN, RMSSD, TRI, VLF, LF, HF, LF/HF, SD1, and SD2 was found between the P^+^ and P^−^ procedure.

#### 3.2.4 Sedation

Not more than one additional dose of detomidine was given to a mare. An additional dose was administered to four mares in replicate 1, to one mare in replicate 2, and to one mare in replicate 3. The median time an additional dose was given was at 24 min after T_0_ (range: 14–41 min). There was a significant effect on sedation found between P^+^ en P^−^ procedures with detomidine doses being significantly higher during P^+^ procedures. The estimated effect of LSM for P^+^ was 55 μg detomidine/100 kg/min ± 0.052 and for P^−^ was 39 μg detomidine/100 kg/min ± 0.077.

## 4 Discussion

The objective of this study was to evaluate the impact of the manipulations characteristic to the TVA procedure, as applied in clinical practice, on pain and stress in mares. We aimed to replicate the conditions of a commercial TVA program by ensuring a sufficient number of follicles for aspiration and scheduling procedures at two-week intervals. The number of follicles aspirated and the oocyte recovery rate aligned with clinical studies reporting an average of 14–26 aspirated follicles per procedure and oocyte recovery rates of 50–70% (2, 54–57). The sample size of 8 mares and the age range (5–18 years) may introduce variability in the results. Despite the age differences, it is important to note that each mare served as their own control. To address this, we have considered individual variances in our analysis by using mixed linear models. This method allows us to build a separate model for each horse, ensuring the consideration of individual variations. Nevertheless, future research with larger sample sizes could further enhance the robustness of these findings.

Our findings revealed that puncturing during TVA (P^+^) did not significantly affect serum metabolite levels nor facial pain scores over the complete procedure. Additionally, HR did not increase at the time of puncturing, and no changes in HRV parameters were observed, when assessed from the insertion up to the removal of the intravaginal device to isolate the effect of puncturing. However, for serum metabolite levels and facial pain scores, differences were observed between specific timepoints and could indicate an effect of puncturing on the course. Despite this, no significant differences were observed between P^+^ and P^−^ at individual timepoints, except for serum glucose levels (T_10_, T_20_, T_40_) and facial pain scores (T_30_).

During restraint in stocks and perineal preparation, serum cortisol and lactate levels and facial pain scores significantly increased in both P^+^ and P^−^. Restraint and preparation were also associated with an estimated increase in HR of 22.6 bpm. These responses may reflect an anticipatory stress response, because the mares were already familiar with the procedure and the associated manipulations. In this sense, it would be valuable to study naïve mares to determine whether similar responses occur without prior experience. However, during the manipulations in the P^−^ procedure (from T_0_ onwards), we observed significant increases in serum cortisol and glucose levels and facial pain scores compared to T_−10_. This aligns with findings from Schönbom et al. (22), who observed a rise in salivary cortisol following transrectal examination. These results suggest that restraint in stocks, preparation of the perineum, insertion of an intravaginal device and rectal manipulation cause an activation of the HPA and SAM axis, indicating not only puncturing itself but all manipulations induce discomfort in mares. Importantly, facial pain scores, HR, and serum metabolite levels returned to baseline after the procedure as measured on Day 1 and 3, suggesting the observed stress response is acute.

The anticipatory stress response, observed by increased serum cortisol and lactate levels, elevated facial pain scores and higher HR at T_−10_ compared to stable measurements, may arise from the mares associating the preparation routine with the TVA procedure itself. This is in accordance with the stress responses documented in horses during activities like loading onto a transport vehicle or being groomed and saddled before riding (58, 59). However, the supposed anticipatory stress response is not reflected in glucose levels in the P^+^ and P^−^ procedure. While the control procedure was conducted last, potentially affecting the anticipatory response, this is unlikely as the mares in this study had undergone multiple TVA procedures prior to the study. This suggests they were already familiar with the procedure, reducing potential confounding effects related to procedural novelty. However, future studies should investigate the cumulative impact of repeated TVA procedures over time.

In contrast to our findings, Diego et al. (23) reported that pain induced by grabbing the ovary and pulling the ligament appeared to be controlled under their experimental conditions, despite using similar medication but without flunixin meglumine. They observed no significant differences in salivary cortisol levels, respiratory rate, or facial expressions between the TVA and control groups, and neither during the TVA procedure overall. Their control group was prepared for the TVA procedure (emptying of the rectum, cleaning of the perineum and placement of a urinary probe) but no intravaginal device was inserted and no rectal manipulations were performed. It is worth noting that they did not measure basal salivary cortisol levels, so it is possible that the mares in their study experienced an anticipatory stress response, as their salivary cortisol levels measured before entry were already elevated above typical resting levels of 0.06–0.11 μg/dL (35). They also suggested that the lack of difference in cortisol between the groups could be attributed to a delay in the detection of salivary cortisol concentrations.

Our findings contribute valuable insights, advancing the current knowledge in this area. However, the monitored parameters may be influenced by some of the administered medications, but administration of medication is intrinsic to this procedure and cannot be left out due to ethical reasons. For instance, butylscopolamine is known to induce tachycardia, while butorphanol and detomidine are both associated with bradycardia, with detomidine additionally causing hyperglycemia (60–62). In horses, constant rate infusion of butorphanol has also been reported to decrease cortisol concentrations (63), and a more recent study in calves found that flunixin meglumine reduces plasma cortisol levels (64). However, the same dosages, adjusted per body weight, were administered to all mares in each procedure, except for detomidine. Therefore, any potential effects on the results between P^+^ and P^−^ due to the administration of butylscopolamine, butorphanol, and flunixin meglumine were likely minimal. A control group undergoing TVA without medication would reflect the ideal positive control to determine the effect of the different aspects of the procedure on pain and stress from a scientifical point-of-view. However, this is ethically not feasible and it was also not the scope of our study.

The detomidine dosage used in P^+^ procedures was significantly higher compared to P^−^ procedures. While the initial dosage of detomidine (15 μg/kg) was standardized for all mares, additional doses (5 μg/kg) were administered as needed to minimize excessive movement that interfered with manipulator operability. Most physiological and behavioral parameters showed no significant differences between P^+^ and P^−^ procedures at any specific timepoint. Importantly, no additional doses of detomidine were administered between T_−10_ and T_10_, suggesting that any difference in parameters observed between P+ and P- during these timepoints cannot be ascribed to variations in detomidine dosage. At these specific timepoints (T_−10_, T_0_, T_10_), no significant differences were observed between P^+^ and P^−^ for most parameters, except for glucose levels, which were elevated at T_10_. Additionally, significantly higher glucose levels in P^+^ at T_20_ and T_40_ observed may be attributed to the higher dosage of detomidine administered after T_10_. Importantly, despite sedation, all procedures showed clear indicators of pain and stress. This implies that, while sedation may effectively provide chemical restraint and suppress expressions of stress during TVA, it may not fully alleviate discomfort from manipulations inherent to the procedure. These findings align with Wilson et al. (65), who observed that detomidine infusion provides prolonged chemical restraint in standing horses but insufficient analgesia for surgical interventions.

The EQUUS-FAP scoring system evaluates pain in horses using 9 facial expression parameters, each scored from 0 to 2 points. A score of 4 points or higher indicates the presence of pain, while a score of 6 points or higher signifies severe pain (30). During the TVA procedure, mares’ scores averaged 7, ranging from 0 to 12, indicating severe pain during the procedure. However, a key limitation is the lack of a validated facial pain scoring system for sedated horses. This makes it challenging to determine whether observed facial expressions reflect pain or sedation. The FaceSed score (66) has shown that sedation significantly affects facial features, such as ear position, orbital opening, and lip relaxation, potentially confounding the assessment of pain with the EQUUS-FAP scoring system. Therefore, these findings should be interpreted with caution. In addition, further studies should aim to develop a pain scoring scale for urogenital pain implementing a checklist to guide sedation and analgesia based on real-time pain evaluations. Importantly, all mares scored a maximum of 3 on Day 1 and 3 after the TVA procedure, showing that the mares experience no pain after the procedure.

Future research could explore alternative methods of pain relief, such as epidural anesthesia or topical gels to relieve the pain experienced when inserting the vaginal probe and to alleviate the tension on the rectal sphincter and hindgut during rectal manipulation. Epidural anesthesia is already standard practice in some equine reproductive clinics, while others use it only selectively. However, recommended dosage of epidural anesthesia desensitizes the anus, rectum, perineum, vulva, vagina, urethra, and bladder, but not the ovaries and ligaments (67). Administration of larger dilutional volumes of drugs into the epidural space may facilitate cranial migration of the drugs but would also cause ataxia or general discomfort in the horse (68). A comparative study examining the effects of TVA with and without epidural anesthesia on pain and stress in mares would be valuable. Although Petyim et al. (18) found that in cattle epidural anesthesia itself caused more stress than the TVA procedure, this area still warrants further investigation. Similarly, the effect of topical gels, like those used in human medicine to reduce pain during intrauterine device insertion, could also be studied. While McNicholas et al. (69) observed no significant reduction in pain with lidocaine gel during intra-uterine device insertion in women, testing various topical gels in mares may yield different results.

In conclusion, the present study investigated the physiological and behavioral responses of mares to the TVA procedure, by evaluating HR, HRV, facial expression changes, glucose, lactate, and cortisol levels before, during, and after the procedure. Restraint in stocks and preparation for the procedure were associated with significant stress, indicating an anticipatory stress response. Signs of discomfort, evidenced by elevated physiological and behavioral parameters, were observed during rectal and vaginal manipulations, while the effect of puncturing itself was minimal. These effects were acute, with all parameters returning to baseline the following day. Although this study established a solid foundation for quantifying pain and stress in mares during the TVA procedure, further research is needed. Efforts should focus on refining the TVA procedure, developing improved pain management protocols, and exploring innovative technologies for more accurate pain and stress assessment to enhance equine welfare.

## Data Availability

The raw data supporting the conclusions of this article will be made available by the authors, without undue reservation.
